# Ionic Liquid-Based Green Emulsion Liquid Membrane for the Extraction of the Poorly Soluble Drug Ibuprofen

**DOI:** 10.3390/molecules28052345

**Published:** 2023-03-03

**Authors:** Huma Warsi Khan, Amal A. M. Elgharbawy, Mohamed Azmi Bustam, Masahiro Goto, Muhammad Moniruzzaman

**Affiliations:** 1 Department of Chemical Engineering, Universiti Teknologi PETRONAS, Seri Iskandar 32610, Malaysia; 2 International Institute for Halal Research and Training (INHART), International Islamic University Malaysia, Kuala Lampur 53100, Malaysia; 3 Center of Research in Ionic Liquids, Universiti Teknologi PETRONAS, Seri Iskandar 32610, Malaysia; 4 Department of Applied Chemistry, Graduate School of Engineering, Kyushu University, 744, Moto-oka, Fukuoka 819-0395, Japan

**Keywords:** ionic liquids, biologically active drug, COSMO-RS, extractant, emulsion liquid membrane, green process

## Abstract

Ibuprofen (Ibf) is a biologically active drug (BADs) and an emerging contaminant of concern (CECs) in aqueous streams. Due to its adverse effects upon aquatic organisms and humans, the removal and recovery of Ibf are essential. Usually, conventional solvents are employed for the separation and recovery of ibuprofen. Due to environmental limitations, alternative green extracting agents need to be explored. Ionic liquids (ILs), emerging and greener alternatives, can also serve this purpose. It is essential to explore ILs that are effective for recovering ibuprofen, among millions of ILs. The conductor-like screening model for real solvents (COSMO-RS) is an efficient tool that can be used to screen ILs specifically for ibuprofen extraction. The main objective of this work was to identify the best IL for the extraction of ibuprofen. A total of 152 different cation–anion combinations consisting of eight aromatic and non-aromatic cations and nineteen anions were screened. The evaluation was based upon activity coefficients, capacity, and selectivity values. Furthermore, the effect of alkyl chain length was studied. The results suggest that quaternary ammonium (cation) and sulfate (anion) have better extraction ability for ibuprofen than the other combinations tested. An ionic liquid-based green emulsion liquid membrane (ILGELM) was developed using the selected ionic liquid as the extractant, sunflower oil as the diluent, Span 80 as the surfactant, and NaOH as the stripping agent. Experimental verification was carried out using the ILGELM. The experimental results indicated that the predicted COSMO-RS and the experimental results were in good agreement. The proposed IL-based GELM is highly effective for the removal and recovery of ibuprofen.

## 1. Introduction

Ibuprofen (Ibf)—chemical formula C_13_H_18_O_2_—is a nonsteroidal anti-inflammatory drug (NSAID), listed as a contaminant of emerging concern (CECs) [1,2]. It is the world’s third most consumed medication [3]. It is used for treating arthritis and musculoskeletal disorders in adults and children [4]. Ibf, when disposed of through various means such as from hospitals, manufacturing industries, excretion processes, etc., undergoes several reactions resulting in more hazardous compounds than the parent drug [5]. The concentration of Ibf in different aqueous streams is greater than 1000 ng/L and poses a great risk to the environment [6]. For the removal of Ibf, commonly used methods include liquid chromatography–mass spectrometry (LC–MS), high-performance liquid chromatography (HPLC) [7], solid-phase extraction (SPE) [8], dispersive liquid–liquid microextraction [9], ultrafiltration, nanofiltration [10], activated carbon [11], etc. These methods employ toxic solvents such as hexane, heptane, etc. [12], that are hazardous to the environment. Hence, treatment technologies with greener solvents and improved extraction efficiencies are required. 

The liquid membrane technology (LMT) has emerged as an efficient extraction technique [13] for the removal of organic pollutants [14], metals [15], and biomolecules [14,16]. LMs include four types: supported liquid membranes (SLM), bulk liquid membranes (BLM), polymer inclusion membranes (PIM), and emulsion liquid membranes (ELM). Although SLM, BLM, and PIMs are effective, they possess low efficiency, are unstable, have a short lifetime, are expensive, and have a low selectivity. The emulsion liquid membranes (ELM), because of their enhanced removal efficiency, increased mass transfer rate, and simultaneous stripping and extraction, are suitable for removing Ibf [17]. The stability of the emulsion is an important factor that governs the potential of ELM. The only issue arising when using this technique is the instability of the emulsion [18]. Therefore, an external agent, also called an extractant, is required [19]. With the increase in environmental awareness and the introduction of the sustainable development goals (SDGs) [20] and green innovative practices [21], in order to follow the fifth principle of green chemistry [22], sustainability [23], and ISO 140001 [24], more focus is being paid to incorporating greener alternatives [25] and green innovative practices (GIPs) [21]. Owing to the outstanding properties and various applications of ionic liquids (ILs) in the extraction [26] and pharmaceutical industries, such as for chemical synthesis, drug delivery [27], and in separation processes [28], it is believed that the use of ILs as carriers in ELM would be beneficial. 

Ionic liquids (ILs), also called “eco-friendly” or “greener” [29] organic solvents, can be incorporated as carriers to remove Ibf. As compared to conventional solvents such as napthenic acids, D_2_EHPA, etc., ILs are characterized by low vapor pressure, minimal solvent losses, thermal stability, and better bonding abilities and hence are greener and better extractants than other solvents. The synthesis of thousands of ILs has been carried out for extraction purposes. However, the extraction of Ibf using IL-based ELM has not been described. Table S1 shows ILs that are being used for the removal of Ibf using various methods. The literature reveals that the most commonly used ILs are imidazolium, phosphonium, and ammonium. However, it is possible to further select ILs with better extraction efficiencies and thermodynamic properties. Since there are many cations and anions forming ILs, it is a cumbersome task to select ILs with excellent extraction properties [18] experimentally. An essential alternative to avoid this tiresome task is a conductor-like screening model for real solvents (COSMO-RS). This is a simulation tool that predicts the activity coefficient at infinite dilution (AC^id^), which is used to evaluate ILs capacity, selectivity, and performance index [30]. As compared to UNIFAC and UNIQUAC, which require large experimental data, COSMO-RS requires only the molecular structure to evaluate the potency of any solvent for a solute under study; hence, it is useful to select solvents with better properties. Furthermore, COSMO-RS provides real insights into the interaction between solute and solvent [31,32]. These predictions are helpful and proved useful for the removal of phenol [33], lactic acid [34], ethenzamide [35], camphorsulfonate [36], aldehydes and ketones [37], propane and propylene [38], and salicylic acid [39].

So far, there have been limited attempts to screen ILs as extractants for emulsion liquid membranes for Ibf separation using COSMO-RS. This is the first study in which a detailed screening using various combinations of ILs for Ibf was performed, to the best of our knowledge. Hence, the main objective of this work was to identify the best cation–anion combination to be used in a potential IL for the removal of Ibf from aqueous streams. The identified IL can be used as an extractant with different extraction techniques. Since there is no research on IL-based ELM applications for Ibf extraction, the identified IL was used in this work to develop an ionic liquid-based green emulsion liquid membrane (ILGELM) for Ibf extraction. Most of the studies for Ibf extraction were carried out using imidazolium [40] and pyridinium [41] cations and PF_6_^−^, BF_4_^−^, Br^−^, and Cl^−^ anions [42,43]. Hence, eight cations, including cyclic, aromatic, non-aromatic molecules, and 19 anions, both hydrophilic and hydrophobic, were selected, obtaining 152 IL combinations. Table 1 and Table 2 provide the list of the selected cations and anions. AC^id^, capacity, selectivity, solvation energies were determined, and evaluations were made to select the best suitable IL.

## 2. Results and Discussion

**Figure 1:**
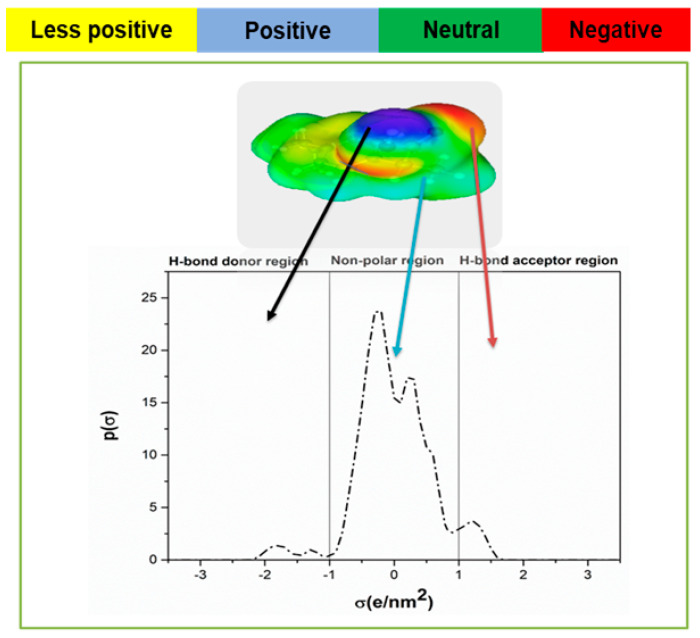
σ-profile of Ibf evaluated using COMSO-RS.

**Figure 2:**
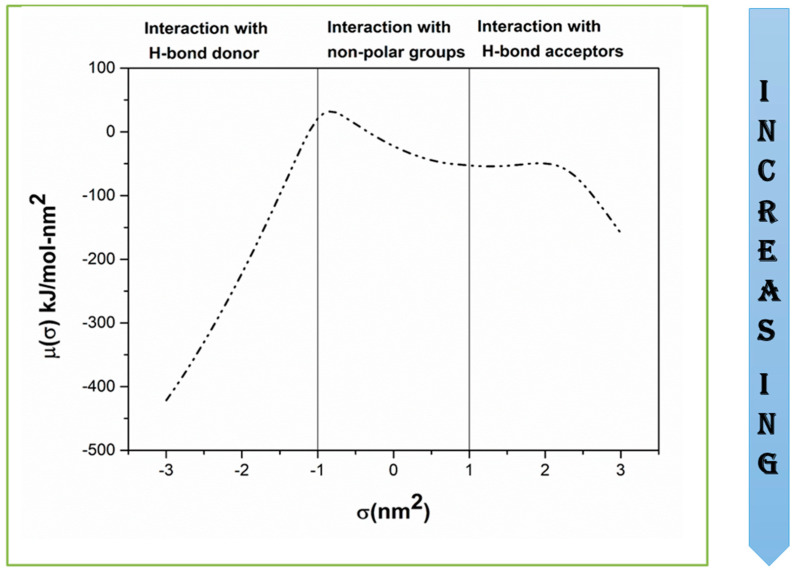
σ-potential of Ibf evaluated using COMSO-RS.

This section presents the results obtained using COSMO-RS for Ibf, the development of the ILGELM using the screened IL, and an experimental validation. Sigma surface along with σ-profile and σ-potential are shown in Figure 1 and Figure 2, respectively. The sigma surface represents the polarity, charge distribution, nature of bonding [39,44]. The colors green, blue, and red represent neutral, positive, and negative charges corresponding to the non-polar, H-bond donor, and H-bond acceptor regions on the σ-profiles [45]. Figure 1 shows sharp and light peaks both in polar and non-polar areas. Ibf shows a sharp peak at 0.3 e/nm^2^ in the non-polar area, revealing its capacity to be more attracted to non-polar molecules. The small peaks in the H-bond acceptor region are due to the presence of the (=O group), and those in H-bond donor regions are due to the OH^−^ group [40]. The presence of peaks in the polar areas signifies an interaction with polar compounds [46]. A small peak is observed at −1.7 e/nm^2^ in the H-bond donor region, and a sharp peak at 1.2 e/nm^2^ in the H-bond acceptor region. As a result, the sigma potential revealed the interaction of Ibf with ILs. Figure 2 indicates that Ibf will function more as an H-bond acceptor (=O group); hence, it will interact and bond well with H-bond donor molecules. The polar nature of Ibf is due to the presence of the carboxylic acid group, and its non-polar nature is due to the presence of benzene and alkyl groups.

### 2.1. Activity Coefficient at Infinite Dilution

**Figure 3:**
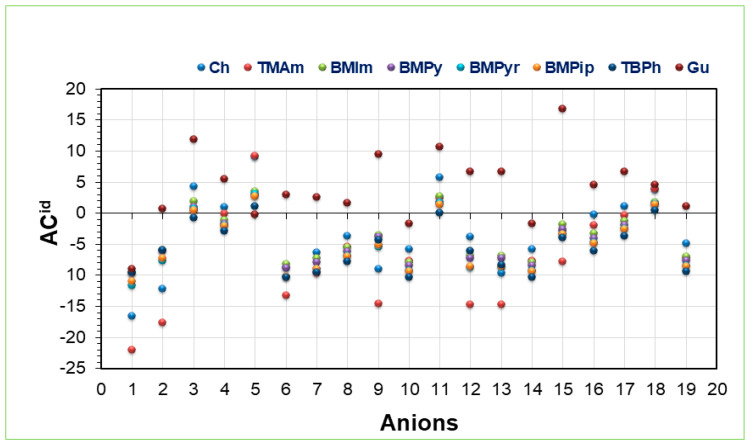
Activity coefficients of the studied ILs for Ibf.

AC^id^ is an important parameter for the preliminary selection of solvents for extraction [47]. The AC^id^ values of 8 selected cations and 19 different anions forming 152 ILs combinations were predicted using COSMO-RS at room temperature. Figure 3 shows the AC^id^ values for different cation–anion combinations screened for Ibf. The lower the value of AC^id^, the better is the IL for separation. The order of cations in relation to the AC^id^ was as follows: [TMAm] < [Ch] < [BMPyr] < [BMPip] < [TBPh] < [BMPy] < [BMIm] < [Gu]. Higher values of AC^id^ are unfavorable for ILs. The results proved that for Ibf, the cations possessing aromatic structures have high AC^id^ values and hence poor extraction capacity for Ibf. This is due to the delocalization of charges caused by pi bonds in ring structures, which imparts stability and poor bonding ability. In contrast, the cations without ring structures, such as ammonium, choline, etc., are favorable for Ibf extraction using an ELM. This is because cation interactions are mainly due to hydrogen bonding between the cation and the heteroatom of Ibf [48]. Table S3 presents the AC^id^ values computed using COSMO-RS.

The anion trend in relation to the AC^id^, as shown in Figure 3, was as follows: SO_4_^2−^ < Cl^−^ < Br^−^ < CH_3_CHOO^−^. The Hofmeister series could well explain this trend. The trend showed that intensely hydrated ions such as SO_4_^2−^, Cl^−^, and Br^−^ possess better extracting ability for Ibf. In contrast, anions such as PF_6_^−^, SCN^−^, and Ntf_2_^−^, which are weakly hydrated, have higher values of AC^id^ and hence are not suitable for Ibf extraction because of their instability [49]. This can further also be explained on the basis of the H-bonding ability of these anions because of their high electronegativity. SO_4_^2−^, Cl^−^, and Br^−^ can form H-bonds quickly with the OH- group of Ibf and hence are better extracting agents for Ibf. In contrast, non-coordinating anions cannot form H-bonds due to their weak bonding nature and thus are unsuitable for Ibf extraction. In addition, anions possessing strong electron-withdrawing fluorinated group results in decreased charge density [50]. These results agree with our previous work on LA showing that quaternary ammonium ILs were potentially useful for its extraction [34]. A similar trend for anions was observed in another study on Ibf where imidazolium-based ILs were studied [40].

### 2.2. IL Capacity towards Ibf

**Figure 4:**
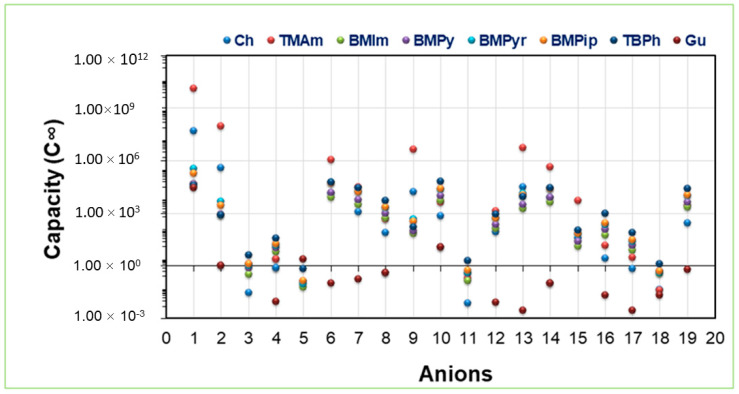
Capacity of the studied ILs for Ibf extraction.

The capacity of the 152 IL combinations was evaluated at 25 °C, and the results are presented in Figure 4. The order of the capacities in relation to cations were [TMAm] > [Ch] > [BMPyr] > [BMPip] > [TBPh] > [BMPy] > [BMIm] > [Gu]. Table S4 presents the capacity values. It was observed that cations consisting of π bonds, i.e., aromatic rings such as pyridinium and imidazolium, showed lower capacity values at infinite dilution (C^∞)^ than cations devoid of it. These results are in good agreement with the results reported in our previous study on LA [34]. Hence, it can be concluded that hydrogen bonding will favor the extraction of Ibf [51].

The anion trend, as shown in Figure 4, was as follows: SO_4_^2−^, Cl^−^, Br^−^, CH_3_CHOO^−^. The trend showed that the SO_4_^2 −^ anion possesses a high capacity compared to other anions. This can be explained as SO_4_^2−^ contains an extra negative charge, making it more suitable for H-bonding than other anions. The other anions that strongly showed higher capacity values were Cl^−^ and Br^−^ This can be explained based on the Hofmeister series indicating the ion specific effect, i.e., that smaller anions possess better H-bonding and are favorable for Ibf extraction. Hydrophobic anions such as PF_6_^−^, SCN^−^, Ntf_2_^−^, which are weakly hydrated and non-coordinating, have lower capacity values and hence are not suitable for Ibf extraction because of their instability [49]. This can also be explained based on the H-bonding ability of these anions. These results agree with work on eicosapentaenoic acid that found that quaternary ammonium ILs were suitable for extraction purposes [52].

### 2.3. Selectivity at Infinite Dilution

**Figure 5:**
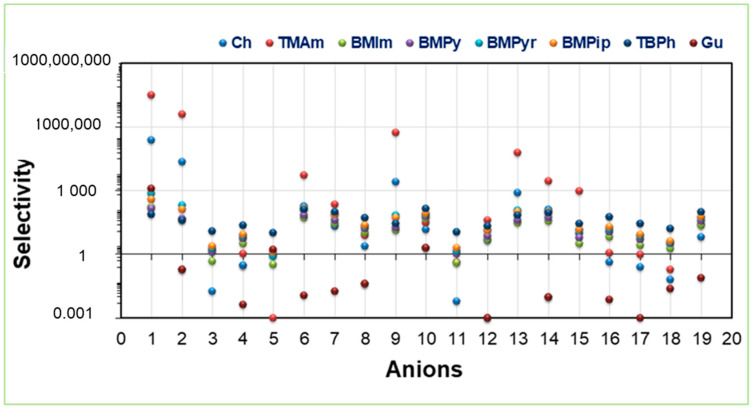
Selectivity of the studied ILs, using COSMO-RS.

Selectivity is an important parameter governing the extraction ability of ILs. Selectivity towards Ibf with ammonium, pyrrolidinium, piperidinium, phosphonium, pyridinium, imidazolium, guanidinium, and choline cations along with 19 anions was estimated via COSMO-RS. The results are present in Figure 5. The following trend was observed for the cations: [TMAm] > [Ch] > [BMPyr] > [BMPip] > [TBPh] > [BMPy] > [BMIm] > [Gu]. Table S5 presents the values of selectivity of the ILs under study. Non-aromatic cations such as ammonium, choline, and pyrrolidinium showed higher selectivity values, hence better extraction ability for Ibf. These cations can form strong H-bonds with Ibf, hence have increased selectivity values. In contrast, aromatic cations showed less selectivity since, because of delocalization and steric hindrance, H-bonding formation was reduced [34].

The anion trend was: SO_4_^2−^, Cl^−^, Br^−^, CH_3_CHOO^−^. The results disclosed that SO_4_^2−^, and Cl^−^ possess the highest selectivity values. The extra negative charge on the SO_4_^2−^ anion is favorable for H-bond formation with Ibf. The Ibf molecule possesses one H-bond donor and two H-bond acceptors. S=O is a good acceptor of protons, forming an H-bond with the Ibf OH^−^ group, thereby improving the solvent’s extraction ability. These results correlate with the results reported for α- docosahexaenoic acid and lactic acid showing that tetramethylammonium sulphate is a potential IL for these BACs [34,53]. 

### 2.4. Performance Index

**Figure 6:**
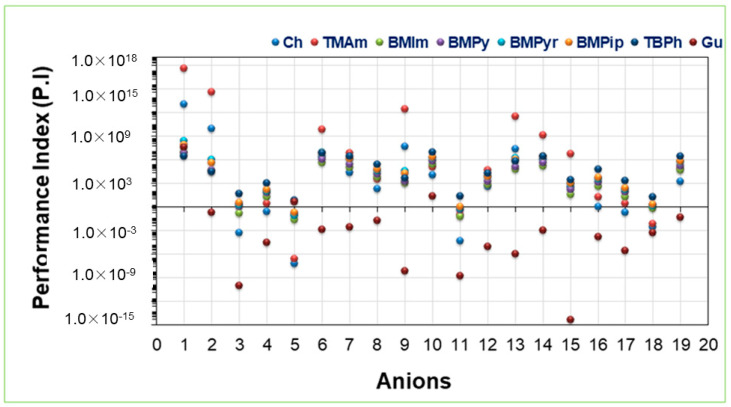
Performance index of the studied ILs for Ibf.

The ILs which possess higher selectivity also have higher values of capacity. Sometimes, ILs that possess high selectivity may not possess high capacities or vice versa. Hence, the performance index is used to find a suitable IL. The performance index is the product of capacity and selectivity. Figure 6 presents the values of [TMAm], [Ch], [BMPyr], [BMPip], [TBPh], [BMPy], [BMIm], [Gu], and 19 anions. The values were calculated using Equation (1). [TMAm], [Ch], and [BMPyr] possess higher values of PI due to their higher values of capacity and selectivity. Table S6 presents the calculated performance index values.

(1) $$\mathrm{PI}=\frac{\gamma_{12}^{\infty }}{\gamma_{12}^{\infty 2}}$$

The highest PI values were observed for [TMAm][SO_4_], [TMAm][Cl], [Ch]S[O_4_], [Ch][Cl], [BMPyr][SO_4_], and [BMPyr][Cl]. It were observed for cations possessing no ring such as [TMAm] and [Ch] and anions with minimum steric hindrance such as [SO_4_^2−^] and [Cl^−^]. 

### 2.5. Solvation Free Energies

**Figure 7:**
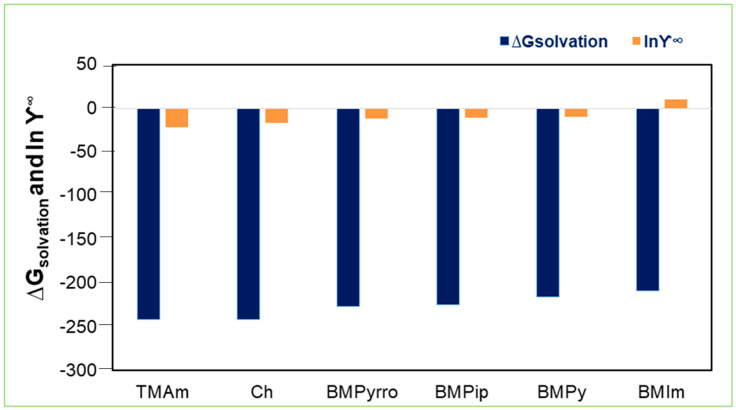
Solvation energies for ILs with the best selected anion.

The solvation energy is related to a molecule’s basic chemical structure in the aqueous phase [54]. The solvation free energies, ∆G_solvation_, for the best cation–anion combinations were estimated using COSMO-RS. The results revealed that SO_4_^2−^ and Cl^−^ possess more negative solvation energy values for all the four cations, with the best solubility values, compared to BF_4_^−^ and PF_6_^−^. These results are in correlation with the AC^id^ values. The IL with the lowest values of AC^id^ and ∆G_solvation,_ is favorable for the extraction of Ibf. Figure 7 shows the values of solvation energy for the SO_4_^2−^ anion. Amongst the cations, the quaternary ammonium cation [TMAm] showed more negative energy values for SO_4_^2−^ and Cl^−^ anions. Hence, these two anions along with [TMAm] will be better extractant for Ibf. In ELM, they can be used as carriers or extractants for increasing the membrane’s stability and efficacy. The ILs as carriers form complexes, promoting the extraction of Ibf from an aqueous solution. The is due to the hydrogen bonding that takes place between Ibf and the ILs [55].

### 2.6. Effect of Alkyl Chain Length upon AC^id^, Capacity, and Selectivity

The effect of alkyl chain length on AC^id^, capacity, and selectivity was studied for the cation–anion combinations for selected anions. As the alkyl chain length increased, AC^id^ increased, resulting in decreased capacity and selectivity. This could be because, as the alkyl chain increased, the hydrophobicity increased due to reduced polarity. It was also observed that ammonium-based cations with a short alkyl chain possessed better values of AC^id^, capacity, and selectivity than those with a longer alkyl chain. As the alkyl chain lengthend, the COSMO volume expanded, resulting in decreased capacity and selectivity [34]. Tables S7 and S8 present the capacity and selectivity values for ILs combinations with alkyl chains. The results suggest that short-chain quaternary-ammonium ILs will be suitable as extractants for Ibf.

### 2.7. Extraction Performance

The ability of extraction for cations and SO_4_^2−^, [Cl^−^], and [BF_4_^−^] anions can further be explained based on the σ-profiles computed using COSMO-RS. Figure S4a presents the σ-profiles for selected anions, i.e., SO_4_^2−^, (2.23 e/nm^2^), Cl^−^ (1.75 nm^2^), and BF_4_^−^ (1.25 e/nm^2^). The results revealed that hydrophobic anions showed a smaller peak and hence less capacity for H-bonding than SO_4_^2−^ and Cl^−^. Figure S1b presents the σ-profiles for the cations under study. The results showed peaks in H-bond donor regions for [TMAm] and [Ch]. In contrast, the peaks for [BMPyr], [BMPip], [TBPh], [BMPy], [BMIm], and [Gu] were in the non-polar region, indicating that these ions possess high Van der Waals affinity; therefore, these cations showed lesser capacity, selectivity, and performance index compared to [TMAm] and [Ch].

### 2.8. ILGELM Extraction for Ibf and Experimental Verification

For the experimental verification of the COSMO-RS results, seven ILs were chosen. Extraction was carried out utilizing an ionic liquid-based emulsion liquid membrane (ILELM). [TMAm][SO_4_], [TMAm][Ac], [TMAm][Cl], [BMIm][Cl], [BMPyrro][Cl], [BMPyrro][Br], and [Ch][Cl] were the ILs selected. In the development of the ILGELM, 0.2 wt.% of IL was employed as a carrier. Table 3 presents the results at various IL concentrations for the ILGELM developed using [TMAm][SO_4_]. The breakage must be less than 10%; however, the lower the breakage the better the performance of the ELM. The results revealed that, in the absence of the IL, the developed ELM was highly unstable, with a high breakage of 5.2%. The addition of the IL improved the stability of the ELM; the ELM was found to be highly stable with 0.25 wt.% of IL ([TMAm][SO_4_]). The breakage was reduced to 1.2%, and the maximum extraction efficiency was obtained. 

### 2.9. COSMO-RS Experimental Validation Using the ILGELM

There are no studies on the AC^id^ values for Ibf–ILs using COSMO-RS. The extraction efficiency obtained using the ILGELM was compared to the AC^id^ predictions determined using COSMO-RS for the specified ILs [32]. The correlation coefficient was computed after plotting the regression curves. The regression curve was also used to calculate the theoretical efficiency. The extraction efficiency was evaluated between theoretical and experimental extractions. The average absolute deviation was determined to validate the results. Figure S5 presents the regression curve for AC^id^ and the extraction efficiency. A linear correlation was found between the predicted AC^id^ values and the experimental efficiencies. The correlation coefficient was found to be approximately 0.96. Using this correlation, the theoretical extraction efficiencies were predicted. The average absolute deviation was found to be 2.9%. The validation of the COSMO tool was performed in our previous work upon the extraction of lactic acid. The predicted results were found to agree with the experimental results, with a deviation of 8.2% [34]. The upper organic phase was recovered and demulsified using a centrifuge. Two layers were formed. The organic layer was emulsified after adding the stripping agent. The formed ILGELM was reused for the extraction of Ibf.

## 3. Materials and Methodology

### 3.1. Materials

Ibf (pure) was procured from PubChem. The sunflower–canola oil blend was purchased from LOTUS supermarket. Sodium hydroxide (NaOH), Span 80, tetramethylammonium acetate [TMAm][Ac] (≥99%), tetramethylammonium chloride [TMAm][Cl] (≥99%), 1-butyl-3-methylimidazolium acetate [EMIm][Ac] (≥99%), 1-Butyl-1-methylpyrrolidinium chloride [BMPyrro][Cl] (≥99%), 1-butyl-1-methylpyrrolidinium bromide [BMPyrro][Br] (≥99%), choline chloride [Ch][Cl] (≥99%), and tributylmethylammonium chloride [TBMAm][Cl] (≥99%) were supplied by Merck, Darmstadt, (Germany). All chemicals used were of analytical grade and were used without further purification. A list of the chemicals used and their properties is present in Table S2.

### 3.2. COSMO-RS Simulation Study

The calculations were performed using COSMOtherm (18.0.2 version, ImbacherWeg, Leverkusen, Germany) software. Considering the molecular structure, COSMO performs geometry optimization and calculates the polarization charged density [39]. Figure S1 shows the step-by-step method for the screening of ILs. The charge density gives an idea of the polarity of the surface and is represented by a histogram. This is further used to evaluate the σ profile and *σ* potential of a molecule. The σ profile and potentials signify the H-bonding nature of the molecule under study [40]. Hydrogen bond energy *E_HB_,* misfit *E_misfit_*, and Van der Waals energy *E_vdW_* are the interaction energies that tell us about the molecule’s bonding nature [40] and can be represented as such [56]. These parameters are expressed by:

(2) $$E_{HB=\alpha_{eff}}\frac{\alpha^{\prime }}{2}\;{\left(\sigma +\sigma^{\prime }\right)}^{2}$$

(3) $$E_{HB=\alpha_{eff}\;C_{HB}\;}\min(0;\min(0;\sigma_{donor\;}+\sigma_{HB)\;max}\left(0;\sigma_{acceptor}-\sigma_{HB}\right)$$

(4) $$E_{vdW}=\alpha_{eff}\left(\tau_{vdW}+{\tau^{\prime }}_{vdW}\right)$$

*α*′ = an interaction parameter

*α_eff_* = the effective contact area

*C_HB_* = strength of hydrogen bonds

*σ_HB_* = hydrogen bonding cut-off

*τ_vdW_* = interaction parameter for specific elements

Using these interaction parameters, the chemical potential is calculated, which is further used to estimate AC^id^. The chemical potential and AC^id^ are evaluated using Equations (5) and (6), respectively

(5) $$\mu_{S}^{X}=\mu_{C,S}^{X}+\int P^{X\;}\;\left(\sigma \right)\mu_{S\left(\sigma \right)}d\sigma$$

(6) $$\gamma =\frac{\mu -\mu_{o}}{RT}$$

where,

*µ* = are the chemical potential of the IL
*µ_o_* = chemical potential of the pure compound

The significance of AC^id^, capacity, and selectivity was highlighted. AC^id^ is an important parameter for the preliminary selection of solvents for extraction [47]. The AC^id^ values are used for the determination of capacity and selectivity. Capacity and selectivity are important for determining the required amount of an IL and governs the extractability. The multiplication of power and selectivity provides the performance index (PI). The lower the value of AC^id^, the higher the value of capacity, selectivity, and PI [28].

(7) $${\left(C_{12}^{\infty }=\frac{1}{\gamma_{1}^{\infty }}\right)}^{IL\;phase}$$

(8) $$\left(\left(S_{12}^{\infty }\right)=\frac{\gamma_{2}^{\infty }}{\gamma_{1}^{\infty 2}}\right)$$

The aqueous phase inherent affinity of Ibf can be found by the evaluation of solvation energies [41].

### 3.3. ILGELM Development

Figure S2 presents the schematic of ILGELM development. An ionic liquid-based green emulsion liquid membrane was prepared using 10 mL of sunflower–canola oil in a conical flask. Span 80, 1 wt.%, and IL, 0.1 wt.%, were added. The resultant mixture was homogenized using a high-speed Ultraturrax homogenizer at 5000 rpm. Then, 2.5 mL of NaOH was added, followed by homogenization at 5000 rpm for 5 min to develop the ILGELM. This ILGELM was added to 25 mL of Ibf (100 µg/L). The solution was stirred using a magnetic stirrer at 240 rpm for 7 min of extraction time. The contents were then poured into a separatory funnel for the separation process to proceed. The solution was allowed to settle for 5 min., resulting in the formation of an upper organic and a lower aqueous phase. The upper phase was the extract phase containing the stripped Ibf, which had been removed from the lower raffinate phase. The upper phase was demulsified to recover Ibf. The lower aqueous phase was filtered, followed by concentration measurement. The concentration of Ibf in the aqueous phase was measured using a UV–vis spectrophotometer at a wavelength of 222 nm, the reference being distilled water. The calibration curve is presented in Figure S3.

The Ibf removal efficiency was calculated using the formula

(9) $$Efficiency\;\left(\%\right)=\left(\frac{C_{o}-C\;}{C_{o}}\right)\times 100$$

*C_o_* = conc. of Ibf in the external phase initially

*C* = conc. of Ibf in the lower aqueous phase after extraction

The stability of ILGELM can be determined by calculating the breakage of ELM. Breakage is defined as when the internal stripping agent spills into the external phase from the membrane phase. Breakage affects the extraction performance and hence is an important parameter. 

Breakage can be calculated using the formula

(10) $$\in \left(\%\right)=\frac{V_{s}}{V_{i}}\;\times \;100$$

where *V_s_* is the amount of stripping agent leaked into the external phase, *V_i_* is the initial amount of stripping agent.

## 4. Conclusions

The recovery and removal of Ibf, an emerging contaminant of concern, using green alternatives are essential in light of the current and future environmental problems. Because of their superior characteristics and eco-friendliness, ILs are a viable alternative to traditional solvents. Since there are millions of ILs, experimentally selecting the best IL is tedious. In addition, the screening of ILs using COSMO-RS for Ibf has not been extensively performed. Hence, COSMO-RS was used to screen suitable IL combinations for the selection of potential ILs. A selected IL was used to develop an ILGELM. The developed ILGELM was applied for the extraction of Ibf. The COSMO-RS results revealed that the combinations of quaternary ammonium cations with SO_4_^2−^ and Cl^−^ anions proved to be the best. This is because of the high electronegativity and better bonding ability of these anions. Non-coordinating anions such as BF_4_^−^ and PF_6_^−^ were not suitable for removing Ibf because of their weak bonding abilities. Furthermore, the developed ILGELM was highly stable and proved efficient for the extraction of Ibf. This research will contribute to the selection of ILs for stable ELMs and other extraction techniques.

## Figures and Tables

**Table 1:** List of the selected cations.

Cation	Acronym
Choline	Ch
Tetramethylammonium	TMAm
1-Butyl-3-methyl-imidazolium	BMIm
1-Butyl-3-methyl pyridinium	BMPy
1-Butyl-1-methyl pyrrolidiniumtoledo	BMPyr
1-Butyl-1-methyl piperidinium	BMPip
Tetrabutylphosphonium	TBPh
Gunadinium	Gu

**Table 2:** List of the selected anions.

Anion	Acronym
Sulphate	SO_4_^2−^
Chloride	Cl^−^
Tetrafluoroborate	BF_4_^−^
Butylsulfate	C_4_H_9_O_4_S^−^
Hexfluorophosphate	PF_6_^−^
Acetate	CH_3_COO^−^
Alaninate	C_3_H_6_NO_2_^−^
Arginitate	C_16_H_13_N_4_O_2_^−^
Bromide	Br^−^
Decanoate	C_10_H_19_O_2_^−^
Perchlorate	ClO_4_^−^
Glutamate	C_5_H_9_NO_4_^−^
Formate	HCOO^−^
Glycinate	C_2_H_4_NO_2_^−^
Nitrite	NO_3_^−^
salicylate	C_7_H_5_O_3_^−^
sachcharinate	C_7_H_5_NO_3_S^−^
bis(trifluoromethyl)sulfonylimide	Ntf_2_^−^
valinate	C_5_H_11_N_2_O_3_^−^

**Table 3:** Breakage and extraction efficiency for Ibf recovery at various IL concentrations.

IL Concentration (wt.%)	Breakage (%)	Extraction Efficiency (%)
0	5.2	28.5
0.1	3.6	56.2
0.15	3.15	64.5
0.2	2.36	81.8
0.25	1.2	93.5
0.3	1.64	87.6
0.35	2.25	78.6

## Data Availability

Not applicable.

## Supplementary Materials

The following supporting information can be downloaded at: https://www.mdpi.com/article/10.3390/molecules28052345/s1, Figure S1 Step by step COSMO-RS methodology to screen ILs for Ibf; Figure S2 Schematic representation of ionic liquid based green emulsion liquid membrane; Figure S3 Calibration curve for Ibf using UV-vis; Figure S4 σ-profiles a)for cations and b) for anions under study; Figure S5 Regression curve for ACid and experimental extraction efficiency; Table S1 ILs used for the removal of Ibf; Table S2 Chemicals and reagents used; Table S3 ACid values predicted using COSMO-RS for ILs; Table S4 Capacity values predicted using COSMO-RS for ILs; Table S5 Selectivity values predictive using COSMO-RS for ILs; Table S6 Performance Index for different ILs; Table S7 Effect of alkyl chain length on the capacity of ILs; Table S8 Effect of alkyl chain length on the selectivity of ILs;

## Abbreviations

[TBPPyri][Br]	4-Tert-butyl-1-propylpyridinium bromide
[EMIm][Trif]	1-Ethyl-3-methylimidazolium trifluoromethane-sulfonate (triflate),
[BMIm][Trif]	1-Butyl-3-methylimidazolium trifluoromethanesulfonate (triflate),
[BMIm][Tos]	1-Butyl-3-methylimidazolium tosylate
[TIBMPh][Tos]	Tri(isobutyl)methylphosphonium tosylate,
[TBPh][Cl]	Tetrabutylphosphonium chloride
[TBAm][Cl]	Tetrabutylammonium chloride
[VBIm][PF_6_]	1-Vinyl-3-butylimidazolium hexafluorophosphate
[BMIm][PF_6_]	1-Butyl-3-methylimidazolium hexafluorophosphate
[BMIm][BF_4_]	1-Butyl-3-methylimidazolium tetrafluoroborate
[OMIm][PF_6_]	1-Octyl-3-methylimidazolium hexafluorophosphate
[D2EHPA]	Di-(2-ethylhexyl)phosphoric acid
