# Supplementary material for: Ionic Liquid-Based Green Emulsion Liquid Membrane for the Extraction of the Poorly Soluble Drug Ibuprofen
Source: Molecules. 2023 Mar 3;28(5):2345. doi: 10.3390/molecules28052345 (PMC10005223; doi:10.3390/molecules28052345)
Supplement: Supplementary file 1 [file molecules-28-02345-s001.zip › molecules-2188038-supplementary.pdf]

Supporting Information

Ionic Liquid-Based Green Emulsion Liquid Membrane for the Extraction of the Poorly Soluble Drug Ibuprofen

Huma Warsi Khan [1], Amal A. M. Elgharbawy [2], Mohamed Azmi Bustam [1,3], Masahiro Goto [4] and Muhammad Moniruzzaman [1,3,*]

[1] Department of Chemical Engineering, Universiti Teknologi PETRONAS, Seri Iskandar 32610, Malaysia
[2] International Institute for Halal Research and Training (INHART), International Islamic University Malaysia, Kuala Lampur 53100, Malaysia
[3] Center of Research in Ionic Liquids, Universiti Teknologi PETRONAS, Seri Iskandar 32610, Malaysia
[4] Department of Applied Chemistry, Graduate School of Engineering, Kyushu University, 744, Moto-oka, Fukuoka 819-0395, Japan
* Correspondence: m.moniruzzaman@utp.edu.my

Table S1 ILs used for the removal of Ibf

S. NO.	**Removal technique**	**IL used**	**Finding**	**Reference**
**1.**	Adsorption	[TBPPyri][Br]	Misfit, hydrogen bond and vander waal energies are responsible the adsorption of Ibf	[41]
**2.**	Aqueous biphasic system (ABS)	[EMIm][Trif] [BMIm][Trif] [BMIm][Tos] [TIBMPh][Tos] [TBPh][Cl]	The addition of ILs increases extraction efficiencies	[43]
**3.**	Solid liquid extraction (SLE)	[TBAm][Cl]	IL results in an improved extraction efficiency of Ibf.	[42]
**4.**	Adsorption	[VBIm][PF_6]	A tremendous increase in Ibf adsorption capacity was observed with the use of IL	[11]
**5.**	Liquid-liquid microextraction	([BMIm][PF_6]) [BMIm][BF_4]	The incorporation of IL enhanced the recovery from 89% to 103%	[2]
**6.**	Dispersive liquid-liquid microextraction	[OMIm][PF_6])	Use of IL resulted in enhanced recovery efficiencies with good enrichment factor.	[9]

Table S2 Chemicals and reagents used

Chemical name	Source	CAS No.	Purity	Impurities
**Sodium hydroxide**	Sigma Aldrich	1310-73-2	>99	<0.5%
**Span 80**	Sigma Aldrich	1338-43-8	-	-
**Tetramethylammonium acetate**	Sigma Aldrich	10581-12-1	>97%	<0.5%
**1-butyl-3-methylimidazolium acetate**	Sigma Aldrich	254049-75-8	>98.0%	<0.5%
**1-butyl-1-methylpyrrolidinium chloride**	Sigma Aldrich	479500-35-1	>99.0%	<0.5%
**1-butyl-1-methylpyrrolidinium bromide**	Sigma Aldrich	93457-69-3<br>93457-69-3	>98.0%	<0.5%
**choline chloride**	Sigma Aldrich	67-48-1	>98.0%	<0.5%
**tributylmethylammonium chloride**	Sigma Aldrich	56375-79-2	>98.0%	<0.5%
**Ibuprofen**	Sigma Aldrich	15687-27-1	>98%	<0.5%
**Sunflower oil**		BILLION Seri Iskandar		

Table S3 AC^{id} values predicted using COSMO-RS for ILs

**S.No.**	**Anions**	**Cations**							
		**Ch**	**TMAm**	**BMIm**	**BMPy**	**BMPyr**	**BMPip**	**TBPh**	**Gu**
**1**	Sulphate	-16.647	-22.112	-9.688	-9.696	-11.624	-11.062	-9.493	-9.066
**2**	Chloride	-12.222	-17.668	-5.971	-6.104	-7.752	-7.286	-6.02	0.7
**3**	Tetrafluoroborate	4.272	0.431	1.829	0.941	0.706	0.423	-0.74	11.82
**4**	Butylsulfate	0.973	-0.152	-1.139	-1.7	-1.975	-2.163	-2.951	5.415
**5**	Hexafluoro Phosphate	9.041	9.155	3.503	2.765	3.123	2.681	1.119	-0.229
**6**	Acetate	-8.784	-13.246	-8.265	-8.873	-10.259	-10.18	-10.309	2.988
**7**	Alaninate	-6.367	-9.667	-7.317	-7.966	-9.033	-9.071	-9.556	2.484
**8**	Argininate	-3.662	-5.475	-5.568	-6.182	-6.867	-7.01	-7.871	1.608
**9**	Bromide	-9.071	-14.612	-3.598	-3.891	-5.451	-5.102	-4.359	9.434
**10**	Decanoate	-5.881	-7.681	-7.892	-8.513	-9.301	-9.438	-10.356	-1.76
**11**	Perchlorate	5.691	2.347	2.622	1.757	1.611	1.293	0.03	10.606
**12**	Glutamate	-3.803	-14.79	-6.842	-7.342	-8.768	-8.585	-6.071	6.637
**13**	Formate	-9.646	-14.79	-6.842	-7.342	-8.768	-8.585	-8.366	6.637
**14**	Glycinate	-5.881	-7.681	-7.892	-8.513	-9.301	-9.438	-10.356	-1.76
**15**	Nitrate	-2.952	-7.886	-1.816	-2.581	-3.478	-3.501	-3.949	16.761

**16**	Salicylate	-0.276	-1.934	-3.336	-4.094	-4.725	-4.964	-6.141	4.538
**17**	Saccharinate	1.052	-0.419	-1.322	-1.972	-2.461	-2.669	-3.652	6.633
**18**	bis(trifluoromethyl) sulfonylimide	3.776	3.931	1.726	1.375	1.514	1.298	0.413	4.596
**19**	Valinate	-4.939	-7.262	-6.973	-7.644	-8.508	-8.634	-9.448	1.143

Table S4 Capacity values predicted using COSMO-RS for ILs

S.NO.	Anions	Cations							
		Ch	TMAm	BMIm	BMPy	BMPyr	BMPip	TBPh	Gu
**1**	Sulphate	5.09E+07	1.28E+10	49201	4958	341619	194652	40448.	2737
**2**	Chloride	406458.	9.42E+07	783.73	894.84	4653.65	2919.2	822.85	0.993
**3**	Tetrafluoroborate	0.028	1.307	0.321	0.781	0.987	1.311	4.193	0
**4**	Butylsulfate	0.756	2.339	6.24	10.944	14.418	17.387	38.255	0.009
**5**	Hexafluoro Phosphate	0.1	0.15	0.0	0.126	0.088	0.137	0.653	2.514
**6**	Acetate	13053.56	1.15E+06	7771.0	14267.74	57096.5	52752.4	59984.45	0.101
**7**	Alaninate	1164.305	32007.2	3009.95	5763.425	16747.2	17391.3	28247.76	0.167
**8**	Argininate	77.841	477.21	523.9	967.549	1921.02	2214.93	5237.896	0.401
**9**	Bromide	17398.4	4.44E+06	73.02	97.878	466.06	328.71	156.387	0
**10**	Decanoate	716.468	4333.646	5349.0	9953.635	21906.1	2510	62865.06	11.625
**11**	Perchlorate	0.007	0.19	0.14	0.345	0.4	0.54	1.941	0
**12**	Glutamate	89.707	1288.	129.7	225.13	518.5	535.7	866.30	0.008
**13**	Formate	30908.	5.30E+	1873	3089.0	12849	10700	8599.8	0.003
**14**	Glycinate	7912.9	44936	4306	7598.9	26609	24728	27012.	0.105
**15**	Nitrate	38.28	5363.	12.28	25.502	64.79	66.26	103.71	0
**16**	Salicylate	2.637	13.82	56.1	119.899	225.37	286.41	929.316	0.021
**17**	Saccharinate	0.699	3.05	7.5	14.372	23.42	28.84	77.126	0.003
**18**	bis(trifluoromethyl) sulfonylimide	0.046	0.03	0.346	0.487	0.421	0.52	1.252	0.02
**19**	Valinate	279.338	2850.4	2134.7	4178.17	9908.2	11237.4	25357.2	0.638

Table S5 Selectivity values predictive using COSMO-RS for ILs

S.No.	Anions	Cations							
		Ch	TMAm	BMIm	BMPy	BMPyr	BMPip	TBPh	Gu
**1**	Sulphate	236778	3.23E+07	170.218	139.77	687.144	379.732	70.445	1219.853
**2**	Chloride	23324	3.77E+06	44.199	45.699	199.761	124.331	38.026	0.185
**3**	Tetrafluoroborate	0.018	1.495	0.452	1.246	1.721	2.376	12.137	0
**4**	Butylsulfate	0.296	1.015	3.026	5.383	6.947	8.479	23.467	0.004
**5**	Hexafluoro Phosphate	0	1.00E-03	0.331	0.88	0.75	1.241	10.19	1.562
**6**	Acetate	178.019	5391.728	50.629	68.02	155.28	138.358	121.92	0.011
**7**	Alaninate	20.801	226.362	27.538	40.09	71.19	71.68	95.11	0.017
**8**	Argininate	2.401	7.35	10.002	15.20	20.69	23.535	51.1	0.039
**9**	Bromide	2525.72	531171	13.282	17.12	69.15	49.144	27.61	0
**10**	Decanoate	14.436	30.422	45.806	60.76	74.51	81.633	144.6	1.964
**11**	Perchlorate	0.006	0.432	0.387	1.07	1.39	1.986	11.074	0
**12**	Glutamate	4.286	38.58	4.639	7.04	12.73	13.017	21.02	1.00E-03
**13**	Formate	752.193	62045.3	30.557	40.73	115.63	94.14	68.94	0
**14**	Glycinate	122.235	2888.7	36.466	49.75	109.80	99.09	90.79	0.009
**15**	Nitrate	8.8	962.1	2.985	5.93	13.371	13.81	26.565	0
**16**	Salicylate	0.429	1.17	6.154	10.97	14.46	18.22	57.293	0.007
**17**	Saccharinate	0.243	0.91	2.599	4.81	7.03	8.71	27.1	1.00E-03
**18**	bis(trifluoromethyl)	0.062	0.187	1.804	3.12	3.22	4.16	15.40	0.022

	sulfonylimide								
**19**	Valinate	6.125	24.82	23.619	34.92	49.62	54.58	98.82	0.076

Table S6 Performance Index for different ILs

S.No.	Anions	Cations							
		**Ch**	**TMAm**	**BMIm**	**BMPy**	**BMPyr**	**BMPip**	**TBPh**	**Gu**
**1**	Sulphate	1.21E+13	4.13E+17	8.38E+06	6.93E+06	2.35E+08	7.39E+07	2.85E+06	3.34E+07
**2**	Chloride	9.48E+09	3.56E+14	34640.74	40893.49	929616	362958.6	31290.14	0.1838
**3**	Tetrafluoroborate	4.94E-04	1.95292	0.14516	0.97266	1.698	3.11382	50.88569	8.24E-11
**4**	Butylsulfate	0.22378	2.37511	18.90308	58.90791	100.16	147.419	897.7335	3.14E-05
**5**	Hexafluoro Phosphate	6.38E-08	2.31E-07	0.01992	0.11084	0.0668	0.16993	6.6539	3.92796
**6**	Acetate	2.32E+06	6.20E+09	393441.6	970497.8	8.87E+06	7.30E+06	7.31E+06	0.00114
**7**	Alaninate	24219.19	7.25E+06	82887.99	231095.9	1.19E+06	1.25E+06	2.69E+06	0.00282
**8**	Argininate	186.8633	3507.686	5239.811	14713.5	39753	52128.4	267707	0.01558
**9**	Bromide	4.39E+07	2.36E+12	969.9269	1676.12	32228	16154	4318.61	7.50E-09
**10**	Decanoate	10342.77	131839	245020.7	60484	1.63E+06	2.05E+06	9.09E+06	22.83731
**11**	Perchlorate	4.19E-05	0.08259	0.05625	0.369	0.55563	1.08967	21.4914	1.80E-09
**12**	Glutamate	384.4966	49711.12	601.9161	1586	6605.931	6973.435	18215	7.88E-06
**13**	Formate	2.32E+07	3.29E+11	57238.88	125828	1.49E+06	1.01E+06	592878	9.61E-07
**14**	Glycinate	967241.7	1.30E+09	157036.5	378104	2.92E+06	2.45E+06	2.45E+06	9.90E-04
**15**	Nitrate	336.8822	5.16E+06	36.68786	151.46	866.4247	915.2552	2755.2	3.77E-15
**16**	Salicylate	1.13124	16.25055	345.7882	1315.558	3259.764	5220.798	53243	1.56E-04
**17**	Saccharinate	0.16981	2.80126	19.49564	69.21772	164.7901	251.4512	2090.1	2.68E-06
**18**	bis(trifluoromethyl)	0.00283	0.00715	0.62352	1.52266	1.35931	2.17617	19.289	4.57E-04

	sulfonylimide								
**19**	Valinate	1711.079	70766.5	50420.43	145915.3	491731	613407.9	2.51E+06	0.04858

Table S7 Effect of alkyl chain length on the capacity of ILs

Cations with varying alkyl chain	SO_4^{2-}	BF_4^-	Cl^-	PF_6^-	CH_3COO^-
Butyltrimethylammonium	623831	0.251795	7035.61	0.022432	26876.85
Tetra-Methylammonium	1.28E+10	1.306643	94200000	2.11E-04	1150711
Tetra-Ethylammonium	2361269	2.455515	29307.39	0.104792	215443.4
Tetra-N-Butylammonium	46169.69	4.356306	924.9798	0.660108	65562.94
Tetrapropylammonium	122488	3.579502	2127.145	0.427954	93126.82
Octyltrimethylammonium	13518.56	0.626098	251.9838	0.163583	8391.253
Methyl-Trioctyl-Ammonium	17416.07	4.409811	417.8525	0.789394	31363.8
Tetradecyltrimethylammonium	5707.758	1.821895	145.8008	0.489927	8074.289
Hexyl-Dimethyl-Isopropylammonium	51818.58	1.241607	889.0637	0.201463	27604.16
1-Hexyl-3-methyl-imidazolium	12877.04	0.555179	253.5241	0.150625	5454.082
1-Butyl-3-ethylimidazolium	26283.51	0.625398	500.0794	0.126039	8217.749
1-Butyl-1-methylpiperidinium	194652.4	1.310575	2919.298	0.136944	52752.43
1-Hexyl-1-methylpiperidinium	51786.71	1.498975	906.2461	0.235345	32065.02
1-Methyl-1-propylpiperidinium	620058.5	1.394286	8408.815	0.095941	85157.97
1-Pentyl-1-methylpiperidinium	94048.66	1.374526	1520.235	0.181949	40616.06
1-Hexadecyl-1-methyl-Piperidinium[2]	12640.47	3.406373	306.7085	0.689067	20992.9
1-Ethyl-1-methyl-Pyrrolidinium	13883000	1.649153	147727.1	0.030103	270434.1
1-Hexyl-1-methyl-Pyrrolidinium	62358.15	1.129228	1019.11	0.173879	30607.61
1-Methyl-1-pentylpyrrolidinium	126886.7	1.005834	1895.63	0.125882	39066.52
1-Methyl-1-propylpyrrolidinium	1494319	1.141635	18337.28	0.056765	105417.2
1-Octyl-1-methyl-pyrrolidinium	26465.07	1.488233	497.9222	0.281652	23241.9
1-Butyl-1-ethyl-pyrrolidinium	325703.6	1.914692	4667.77	0.171112	94258.02
Butyl-trihexyl-phosphonium	33814.22	5.184436	717.5374	0.804386	64042.84
Decyl-trihexyl-phosphonium	31695.48	5.22572	671.4244	0.804366	62390.03
Tetrabutyl-phosphonium	40448.12	4.192579	822.8536	0.652953	59984.45
Tetrahexyl-phosphonium	32616.22	5.24789	695.1285	0.813184	63002.88
Tributyl-ethyl-phosphonium	57649.8	3.924964	1090.038	0.570416	73102.75
Di[N-Dimethyl]-N(butyl,tetradecyl)guanidinium	110507.2	7.01794	1715.96	0.878806	244127

1-Butyl-3-ethyl-pyridinium	26372.66	1.071053	539.1929	0.202725	12307.15
1-Butyl-3-methyl-pyridinium	49589.57	0.780658	894.8488	0.125964	14267.74
1-Hexadecylpyridinium	3991.602	2.519436	123.1845	0.718999	5188.235
1-Hexyl-4-methyl-pyridinium	16446.33	1.148992	354.3297	0.252073	10155.16
4-Methyl-1-octyl-pyridinium	10296.15	1.591048	245.7814	0.388041	9045.078
Guanidinium	27377.82	1.47E-05	0.992824	2.514337	0.10076
Hexamethylguanidinium	15421000	15.00865	155608.2	0.428502	2425178
Di[N-Dimethyl]-N-[butyl,octyl]-guanidinium	98852.36	6.692637	1534.847	0.848473	230458.1
Di[N-Di(2-methoxyethyl)]]-N-dimethyl-guanidinium	210354	5.830743	2937.677	0.953614	250311.2

Table S8 Effect of alkyl chain length on the selectivity of ILs

**Cations with varying alkyl chain**	**SO_4^{2-}**	**BF_4^-**	**Cl^-**	**PF_6^-**	**CH_3COO^-**
Butyltrimethylammonium	1821.1	0.3	357.89	0.14	119.4
Tetra-Methylammonium	32253000.0	1.4	3774875.8	0.0010	5391.7
Tetra-Ethylammonium	3567.6	4.11	1033.6	0.91	430.9
Tetra-N-Butylammonium	78.1	12.4	41.8	10.2	130.6
Tetrapropylammonium	180.03	8.01	81.0	5.2	167.0
Octyltrimethylammonium	49.2	1.2	16.3	1.3	43.0
Methyl-Trioctyl-Ammonium	49.2	17.66	29.2	15.5	108.9
Tetradecyltrimethylammonium	25.2	4.7	11.7	5.60	48.7
Hexyl-Dimethyl-Isopropylammonium	120.3	2.4	43.8	1.9	85.5
1-Hexyl-3-methyl-imidazolium	49.0	0.8	15.9	0.9	38.0
1-Butyl-3-ethylimidazolium	83.5	0.94	27.3	0.7	49.3
1-Butyl-1-methylpiperidinium	379.7	2.37	124.3	1.2	138.3
1-Hexyl-1-methylpiperidinium	111.8	3.08	43.1	2.4	90.3
1-Methyl-1-propylpiperidinium	1132.3	2.3	333.6	0.8	213.6
1-Pentyl-1-methylpiperidinium	192.3	2.6	68.5	1.7	109.1
1-Hexadecyl-1-methyl-Piperidinium	38.6	11.0	21.1	10.7	81.9
1-Ethyl-1-methyl-Pyrrolidinium	24030.9	2.4	5385.4	0.2	675.6
1-Hexyl-1-methyl-Pyrrolidinium	140.2919479	2.248682	49.5	1.06	89.2
1-Methyl-1-pentylpyrrolidinium	271.584757	1.876005	87.02	1.15	110.3
1-Methyl-1-propylpyrrolidinium	2800.053031	1.8	727.9	0.45	274.6
1-Octyl-1-methyl-pyrrolidinium	65.1	3.3	26.6	3.07	72.7
1-Butyl-1-ethyl-pyrrolidinium	531.6	3.6	181.7	1.6	194.1

Butyl-trihexyl-phosphonium	66.1	20.1	39.4	16.7	139.1
Decyl-trihexyl-phosphonium	70.93	25.1	43.2	20.1	154.0
Tetrabutyl-phosphonium	70.4	12.1	38.0	10.1	121.9
Tetrahexyl-phosphonium	67.02394	22.0280	40.424808	18.130	143.64
Tributyl-ethyl-phosphonium	92.8467869	10.47153	46.943426	8.289442	134.881573
Di[N-Dimethyl]-N(butyl,tetradecyl)guanidinium	139.6	31.7	78.2	22.9	268.3
1-Butyl-3-ethyl-pyridinium	74.9	1.8	28.1	1.5	59.2
1-Butyl-3-methyl-pyridinium	139.7	1.2	45.6	0.8	68.0
1-Hexadecylpyridinium	22.8	6.60	11.5	7.5	49.0
1-Hexyl-4-methyl-Pyridinium	50.6	2.089	20.05	1.98	52.2
4-Methyl-1-octyl-Pyridinium	34.38	3.2	15.2	3.3	50.2
Guanidinium	1219.85	0.001	0.18	1.5	0.01
Hexamethylguanidinium	11302.1515	28.97068	3731.613243	5.01703	1868.3
Di[N-Dimethyl]-N-[butyl,octyl]-Guanidinium	136.5	34.8	77.8	25.0	272.5
Di[N-Di(2-Methoxyethyl)]]-N-dimethyl-guanidinium	243.9	15.5	112.51	10.7	274.9

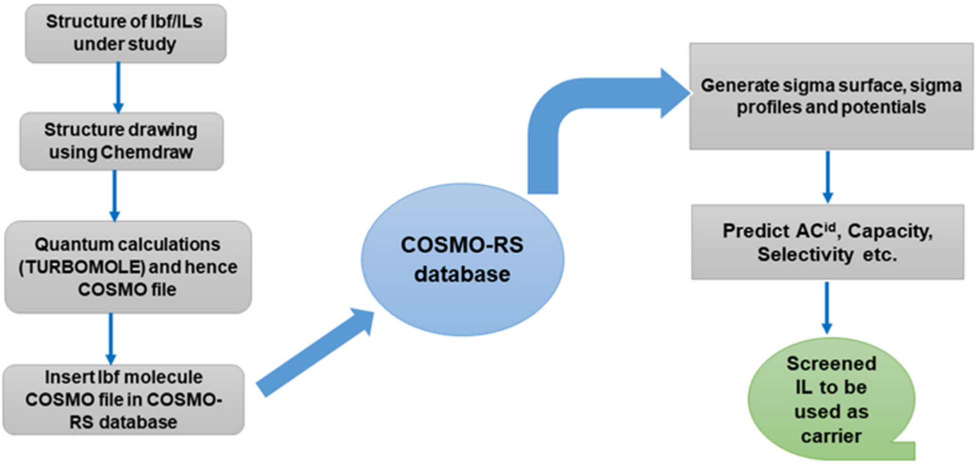

Figure S1 Step by step COSMO-RS methodology to screen ILs for Ibf

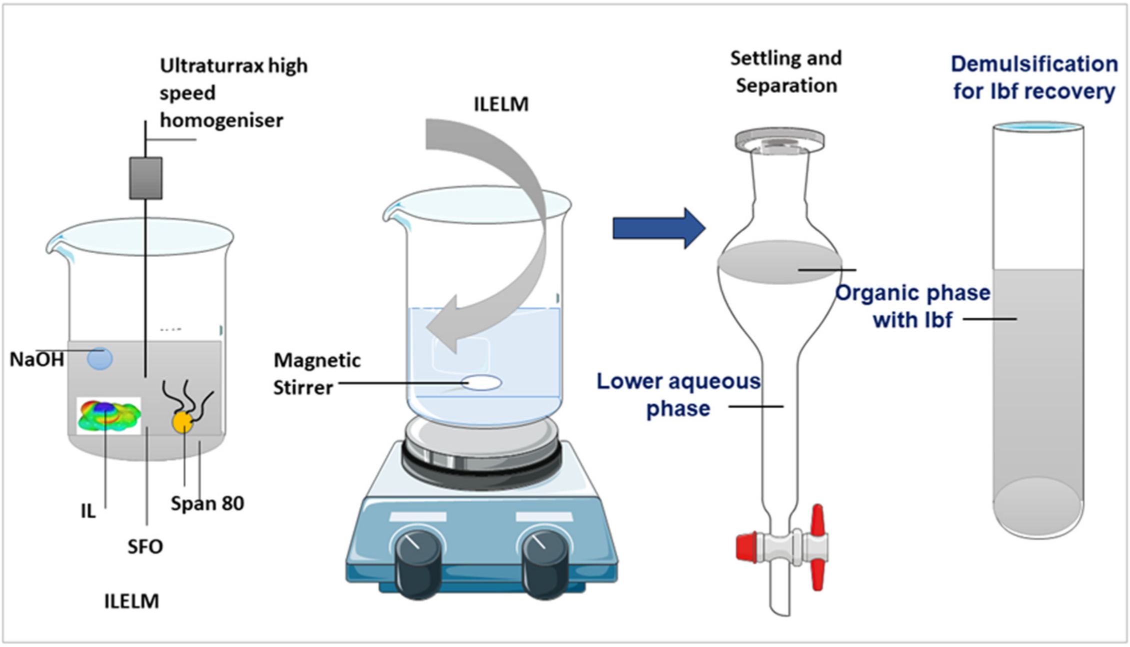

Figure S2 Schematic representation of ionic liquid based green emulsion liquid membrane

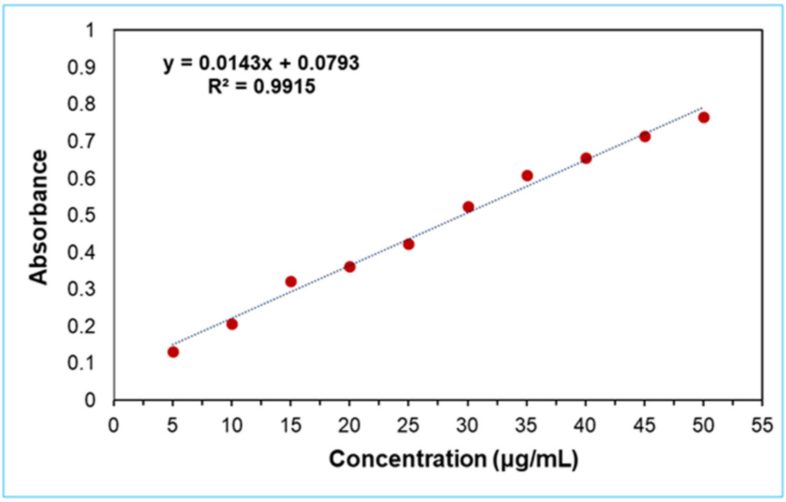

Figure S3 Calibration curve for Ibf using UV-vis

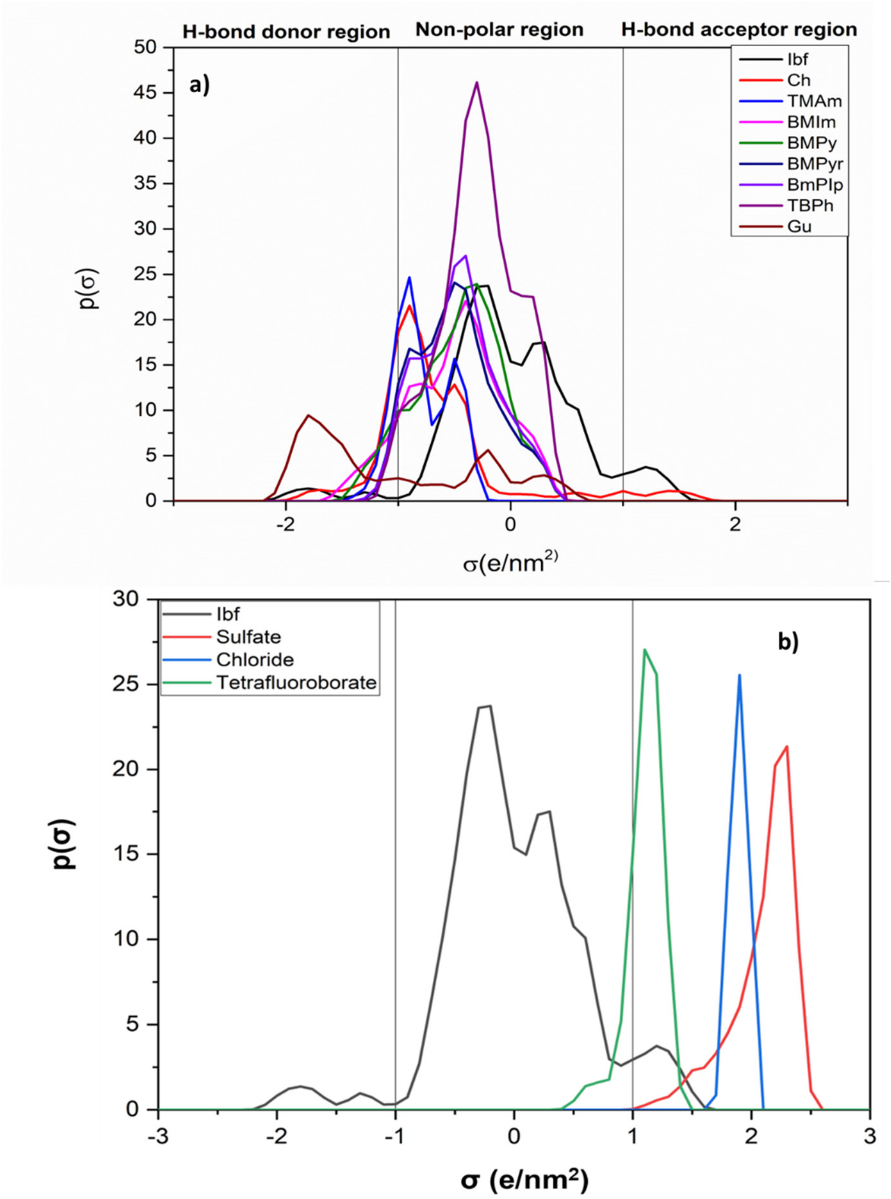

Figure S4 σ-profiles a)for cations and b) for anions under study

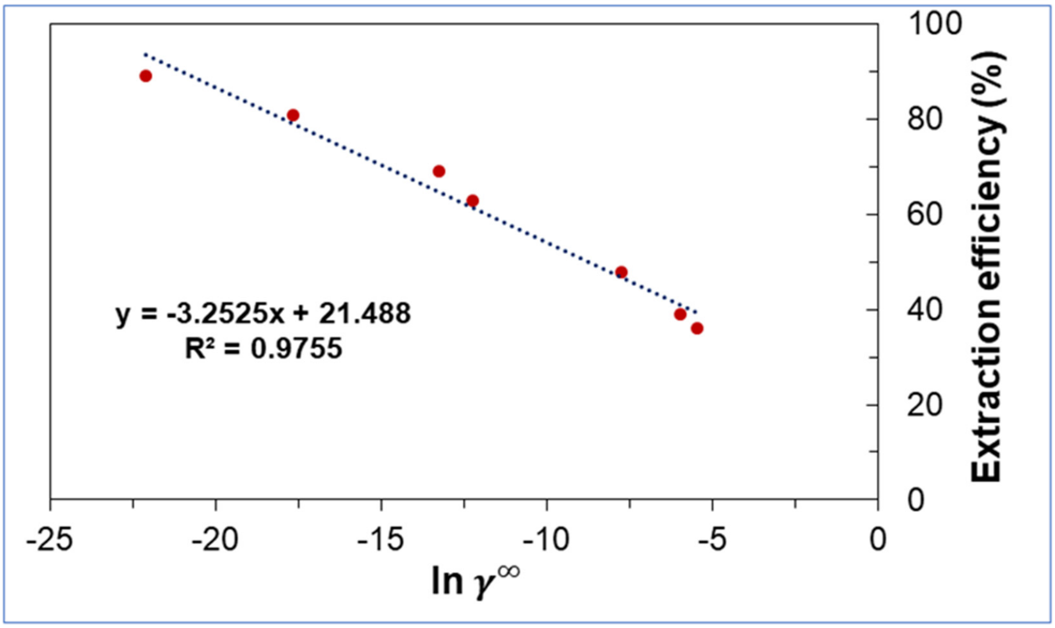

Figure S5 Regression curve for AC^{id} and experimental extraction efficiency
